# Effect of Porosity on Dynamic Response of Additive Manufacturing Ti-6Al-4V Alloys

**DOI:** 10.3390/mi13030408

**Published:** 2022-03-04

**Authors:** Yihang Cui, Jiacheng Cai, Zhiguo Li, Zhenyu Jiao, Ling Hu, Jianbo Hu

**Affiliations:** 1 Laboratory for Shock Wave and Detonation Physics, Institute of Fluid Physics, China Academy of Engineering Physics, Mianyang 621900, China; cuiyihang19@gscaep.ac.cn (Y.C.); zhiguo_li@foxmail.com (Z.L.); huling@ustc.edu (L.H.); 2 School of National Defense Science and Technology, Southwest University of Science and Technology, Mianyang 621010, China; cjc15508005281@163.com; 3 State Key Laboratory for Environmentally Friendly Energy Materials, Southwest University of Science and Technology, Mianyang 621010, China; zhenyujiao1996@163.com

**Keywords:** additive manufacturing, Ti-6Al-4V, dynamic behaviors, porosity, spall

## Abstract

Additive manufacturing is a rapidly developing manufacturing technology of great potential for applications. One of the merits of AM is that the microstructure of manufactured materials can be actively controlled to meet engineering requirements. In this work, three types of Ti-6Al-4V (TC4) materials with different porosities are manufactured using selective laser melting using different printing parameters. Their dynamic behaviors are then studied by planar impact experiments based on the free-surface velocity measurements and shock-recovery characterizations. Experimental results indicate that the porosity significantly affects their dynamic response, including not only the yield, but also spall behaviors. With the increasing porosity, the Hugoniot elastic limit and spall strength decrease monotonically. In the case of TC4 of a large porosity, it behaves similar to energy-absorbing materials, in which the voids collapse under shock compression and then the spallation takes place.

## 1. Introduction

Additive manufacturing (AM) technology has a series of technical advantages such as rapid prototyping, free manufacturing, and high material utilization, thus showing great development potentials and broad application prospects in automation industry [1,2], aerospace [3,4], shipbuilding, biotechnology [5], automobile [6], parts processing [7], and other fields [8,9]. Several critical reviews regarding AM technological innovation processes have been published [10,11,12,13]. Recently, AM technology has shown one advantage which is even more attractive—that is, it can actively manipulate the microstructure of materials by adjusting printing parameters and strategies, such that some unique and excellent mechanical properties could be designed for specific applications. For example, AM components with special microstructures such as honeycomb and gradient can significantly improve heat transfer and anti-collision performance [9,14].

In many engineering cases, the mechanical properties of materials under dynamic loading are of importance and have been approved to be sensitive to the internal microstructure [15,16,17]. Therefore, there is an urgent demand to employ AM technology to control the microstructures, and then to manipulate the macroscopic dynamic properties of materials. By far, only a few works are available to study the dynamic response of AM materials, especially on the effect of porosity produced during printing. For instance, Valdez et al. induced various levels of porosity in Super Alloy 718 by modifying the powder bed fusion process including laser power, scan velocity, and hatch spacing, and then investigated their dynamic behaviors [18,19]. Their results show that, in the presence of high porosity, porous materials perform much like open-cell foams and are highly sensitive to densification. Branch et al. demonstrated shock wave modulation or “spatially graded-flow” in shock wave experiments via controlling AM techniques at the micron scale by using time-resolved X-ray imaging [20]. Gangireddy et al. discovered that porous sandwich Ti-6Al-4V (TC4) AM samples exhibited greater energy absorption per unit volume than fully dense samples using split-Hopkinson Pressure Bar (SHPB) testing [21]. However, there is still a lack of systematic understanding of the effect of porosity on the dynamic mechanical properties of AM materials.

In order to better understand the effect of porosity on the dynamic response, in this work, we prepare three TC4 specimens of different porosities by using different printing parameters in the Selective Laser Melting (SLM) process and then carry out a series of planar impact experiments to investigate their dynamic behaviors. Results demonstrate that dynamic mechanical properties, including the Hugoniot elastic limit and spall strength, are significantly affected by the porosity. A small difference in porosity could remarkably reduce the dynamic properties. Depending on the porosity, the materials may present a good energy absorption capability.

## 2. Material Manufacturing and Characterization

**Figure 1:**
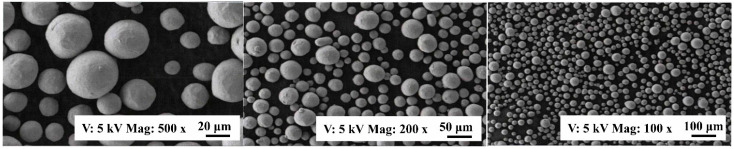
The SEM image of the TC4 powder.

TC4 specimens with different porosities are produced by the SLM process. The particle size of the used powder is 15~45 µm, which was measured by scanning electron microscope (SEM) as shown in Figure 1. The chemical composition of TC4 powder and workpiece is listed in Table 1. The porosity is controlled by adjusting the printing parameters, including scan velocity, laser power, and hatch spacing, as given in Table 2. The printing parameters are chosen based on the Refs. [22,23,24,25]. The stack of layers is along the Z-axis with the layer thickness of 30 μm and the scanning strategy of 45° rotation between layers.

**Figure 2:**
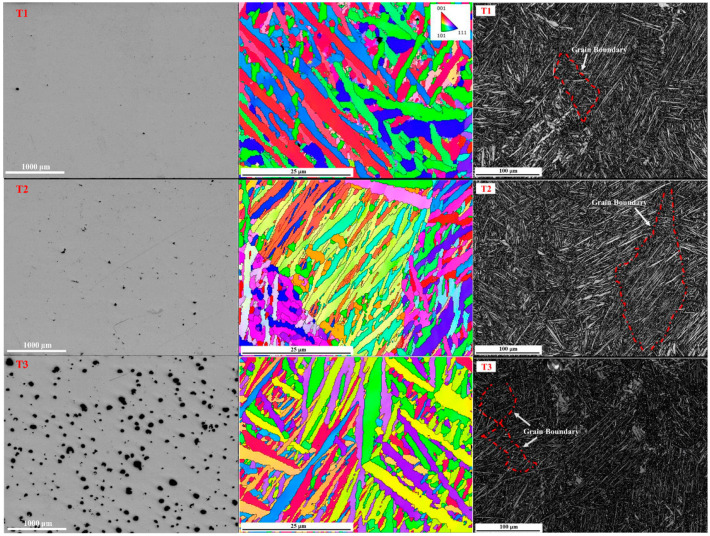
Optical microscopy (**left**), EBSD (**middle**), and band-contrast images (**right**) of the SLM Ti-6Al-4V materials studied. Top view: sample T1 of porosity 0.29%; middle view: sample T2 of porosity 0.88%; bottom view: sample T3 of porosity 5.41%.

To quantify the porosity of the samples, the micro-morphology of three samples is analyzed by optical microscopy (OM), as shown in the left column of Figure 2. It is obvious that the porosity of the three samples increases from T1 to T3. The average pore sizes of T1, T2, and T3 samples are 1.8 μm, 3.2 μm, and 81.5 μm, respectively. Using the drainage method, we determine the density (*ρ*_0_) of each sample and then calculate the porosity according to (1-*ρ*_0_/*ρ_d_*), where *ρ_d_* is the density of dense wrought TC4. As given in Table 2, the samples T1-T3 have the porosity of 0.29%, 0.88%, and 5.41%, respectively.

The inverse pole-figure obtained via electron backscatter detection (EBSD), shown in the middle column of Figure 2, presents needle-shaped textures, indicating the existence of acicular α martensite [26,27]. The size of acicular α martensite for all three samples is almost the same, with an average grain width of 1.9–2.0 μm and a length of 10–100 µm. Further, acicular α martensite forms complex β columnar grains which appear along the building direction with the size of 100s μm. Continuous grain boundaries are observed in three band-contrast images, as marked by the red dotted line shown in the right column of Figure 2 [28,29].

The acoustic velocities (longitudinal wave velocity, *C_L_*, and shear wave velocity, *C_s_*) of each sample are determined by using the pulse-echo method, respectively. Then, the bulk sound velocity, *C*_0_, is calculated by

(1) $$C_{0}=\sqrt{C_{L}^{2}-\frac{4}{3}C_{s}^{2}}$$

All these results are listed in Table 2.

**Figure 3:**
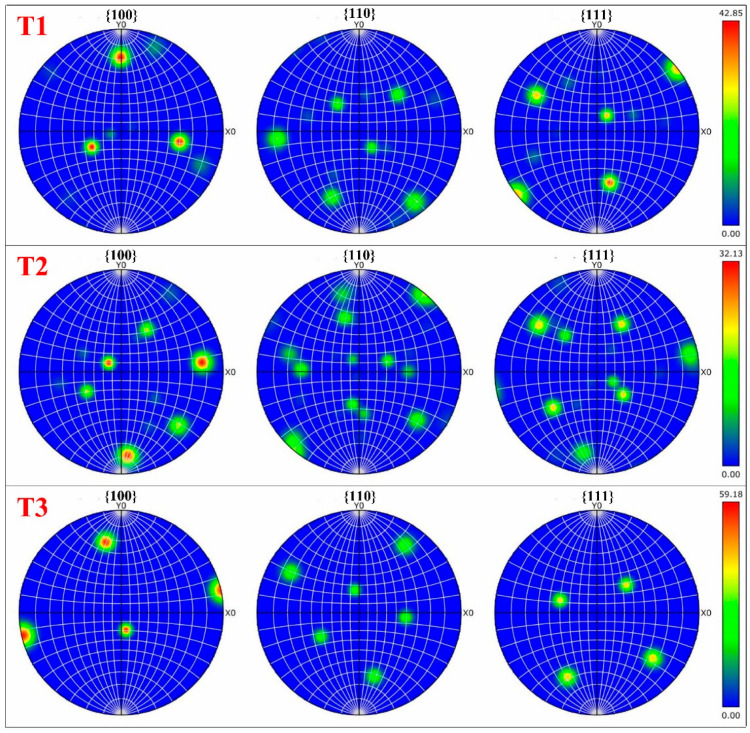
Pole figure maps of SLM Ti-6Al-4V materials studied.

Figure 3 presents the sample textures. It shows that the samples represent a stronger texture in the {100} orientation than that of {110} and {111} orientations. The maximum texture intensities of T1, T2, and T3 samples observed in the {100} orientation are 42.85, 32.13, and 59.18, respectively, indicating that T3 has the most concentrated texture. This is because the change in printing parameters leads to different cooling rates and grain growth mechanisms, resulting in different grain orientations and texture intensities [30].

## 3. Shock-Wave Experiments and Results

### 3.1. Planar Impact Experiments

**Figure 4:**
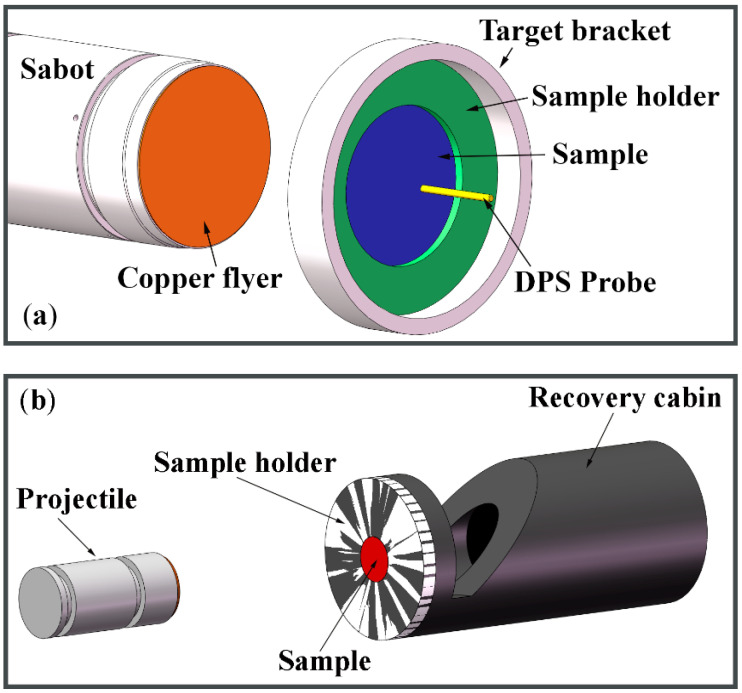
Experimental configurations for (**a**) free-surface particle velocity measurements and (**b**) shock-recovery characterizations. A Cu flyer is launched by a single-stage gas gun to produce spall damage in TC4 samples. DPS is used to monitor the free-surface velocity profile. A recovery cabin is used to capture the target sample for shock-recovery characterizations.

Planar impact experiments were performed on a single-stage gas gun with a caliber of 14 mm. Figure 4a schematically shows the experiment configuration for measuring the free-surface particle velocity history. SLM TC4 samples were wire-cut to 1.8 mm in thickness and 12 mm in diameter for experiments to make sure that the shock compression was along the z-axis. The oxygen-free copper flyer plate was 0.9 mm in thickness to drive tensile damage in the center of the TC4 sample. The impact speeds of the flyer impact are 500 m/s and 620 m/s, respectively. A Doppler Pin System (DPS) was used to probe the free-surface particle velocity [31,32,33]. Figure 4b shows the configuration for shock-recovery experiments. To prevent possible secondary damage during shock recovery, a stainless-steel recovery cabin filled with low-density vacuum-sealing putty was used to capture the target samples after dynamic tensile.

### 3.2. Results and Discussion

**Figure 5:**
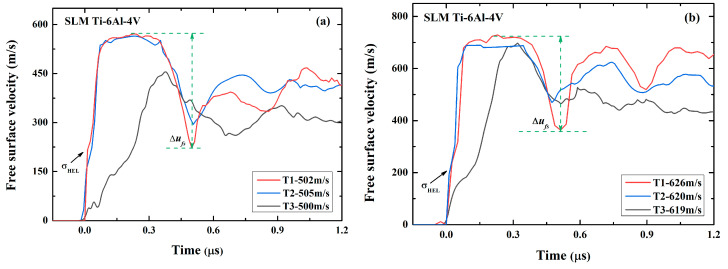
Free-surface particle velocity profiles of three SLM TC4 samples obtained in planar plate experiments at the impact velocity of (**a**) 500 m/s and (**b**) 620 m/s.

**Figure 6:**
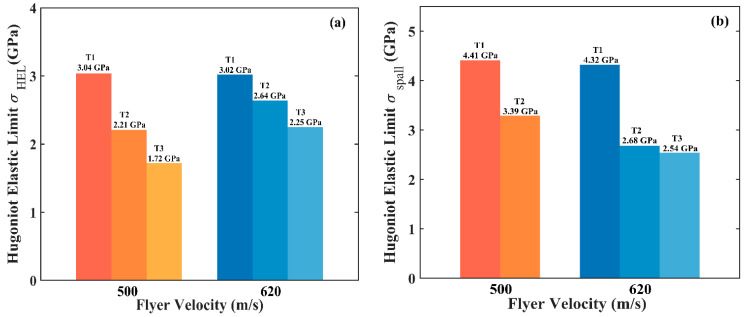
Hugoniot elastic limit (**a**) and spall strength (**b**) of three different TC4 materials produced by SLM. Sample number and loading velocities are indicated on each bar.

Figure 5 presents the free-surface particle velocity profiles for all the samples and at two different impact speeds. For samples T1 and T2, the velocity profiles show an obvious elastic-plastic transition during loading. The Hugoniot elastic limit (*σ_HEL_*) is, thus, calculated by

(2) $$\sigma_{HEL}=\frac{1}{2}\rho_{0}C_{L}u_{HEL},$$

where *σ_HEL_* is the velocity at the transition point. It is clear in Figure 6a that *σ_HEL_*, for the sample T1, is bigger than that of T2, suggesting that an increase in porosity leads to a decrease in *σ_HEL_*. The difference in *σ_HEL_* between the two samples is about 0.38~0.83 GPa (that is, 13%~27%), depending on the impact velocity.

We can also calculate the spall strength (*σ_spall_*) based on the observed ‘pullback’ signal in the free-surface velocity profiles, which is caused by the spallation damage when two rarefaction waves originated from the flyer’s rear free surface and the sample’s free surface, by

(3) $$\sigma_{spall}=\rho_{0}C_{L}\Delta u_{fs}\frac{1}{1+\frac{C_{L}}{C_{0}}},$$

where $\Delta u_{fs}$ is the difference of the free surface velocity from the peak value to the first minima, as indicated in Figure 5. The calculated results are shown in Figure 6b. This demonstrates that, although the porosity difference between the two samples is only 0.59%, the difference in the spall strength is more than 25%. Therefore, the porosity produced during printing significantly affects the dynamic response, including both the yield and spall behaviors. Compared with wrought TC4 (5.28 GPa) [22], the spall strength of SLM TC4 is much smaller. The degradation in the spall strength might be due to the combined contrition of the pores and microstructure. It is well known that the microstructures of SLM and wrought materials could be remarkably different. In this work, however, it is impossible to identify which is playing the dominant role.

For sample T3, the free-surface velocity profile is extremely different from that of samples T1 and T2. The free-surface velocity increases gradually and no shock wave is formed. This is due to the compaction of numerous voids under dynamic compression. Note that, at the impact velocity of 500 m/s, the peak free-surface velocity is remarkably lower than that of the samples T1 and T2, while at 620 m/s the peak velocity is almost the same as the ones in samples T1 and T2. It thus suggests that the voids in the sample T3 are completely compacted and the sample becomes dense at higher shock pressure.

The Hugoniot elastic limit and spall strength of the sample T3, as shown in Figure 6, are calculated by using Equations (2) and (3), respectively. Results demonstrate that, as expected, both *σ_HEL_* and *σ_spall_* of the sample T3 are further lowered due to the large porosity. Based on the above comparison, it is concluded that the increase in porosity markedly reduces the Hugoniot elastic limit and spall strength of SLM TC4 materials, thus degrading their mechanical performance.

Jones et al. have also investigated the spall strength of SLM TC4 [22]. In the case of the loading direction parallel to the printing one which is the same as us, SLM TC4 samples had the spall strengths of 3.03 GPa and 3.34 GPa at the impact velocities of 310 m/s and 415 m/s, respectively. In comparison with our result, we speculate that SLM TC4 materials used in Ref. [22] may have a porosity of more than 0.5%.

**Figure 7:**
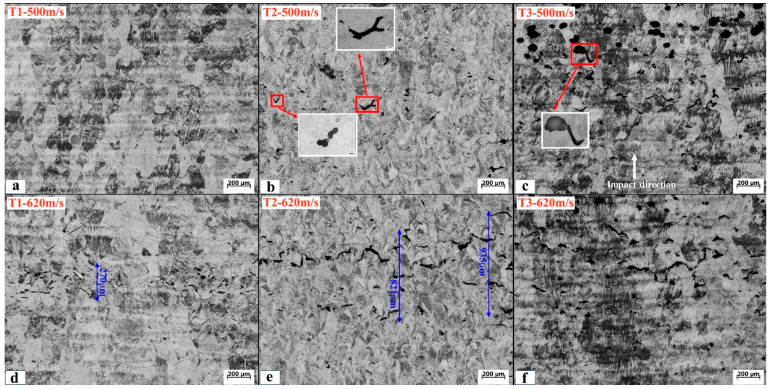
Optical metallography of three SLM TC4 samples shock-recovered at the impact velocity of (**a**–**c**) 500 m/s and (**d**–**f**) 620 m/s.

To further understand the dynamic behaviors of shocked materials, shock-recovered samples were characterized by using OM, as shown in Figure 7. At the impact velocity of 500 m/s, no cracks are visible in sample T1, while in the samples T2 and T3 there exist obvious isolated cracks. Some cracks have rounded tips, suggesting the coalescence of voids [34,35]. Such an observation is somehow inconsistent with the observed velocity profiles in which the spallation occurs in the samples T1 and T2. We attribute this inconsistency to the incomplete spallation in sample T1 because it has a higher resistance to dynamic tension than the samples T2 and T3 as indexed by the spall strength. In the sample T3 recovered, the collapse of voids is clearly observed, providing direct evidence for the shock-driven compaction. At the impact velocity of 620 m/s, complete spallation takes place in all three samples, thus obvious spallation damages are visible. In the sample T1, a large number of small cracks with a length of less than 50 µm locate at the zone with a width of 270 μm. While in the samples T2 and T3, the damage region expands significantly, suggesting that cracks originate from widely distributed voids.

## 4. Conclusions

We have printed TC4 materials with different porosities by designing the printer parameters and experimentally investigated the effect of porosity on the dynamic response of SLM TC4 materials. Experimental results indicate that:

- SLM TC4 with different porosities show observably different dynamic characteristics in the free-surface particle velocity profiles. The increase in porosity significantly degrades the dynamic mechanical properties, including the Hugoniot elastic limit and the spall strength.
- Dense samples show better tensile-resistant properties than porous samples, while porous samples show a good energy absorption capability than dense samples.
- Shock-recovery characterizations indicate that, in porous samples, void collapse and energy absorption occur in the impact stage and cracks mainly originate from widely distributed voids.

Therefore, materials with various dynamic properties can be produced by selecting certain printing parameters. This work may guide AM technology to design materials for certain applications under extreme conditions.

## Figures and Tables

**Table 1:** The chemical composition of TC4 powder and workpiece.

	Element	Al (wt.%)	V (wt.%)	O (wt.%)	N (wt.%)	C (wt.%)	H (wt.%)	Fe (wt.%)	Ti (wt.%)
Condition									
Powder		5.5~6.75	3.5~4.5	<0.2	<0.05	<0.08	<0.015	<0.3	Balance
Workpiece		5.98	4.11	0.12	0.022	0.013	0.010	0.029	Balance

**Table 2:** Summary of SLM parameters and sample’s porosities and physical parameters. C*_l_*, C*_s_*, C*_b_* represent the longitudinal, shear, and bulk acoustic velocities, respectively.

Sample	Power (W)	Scan Velocity (mm/s)	Track Width (mm)	C*_l_* (km/s)	C*s* (km/s)	C*_b_* (km/s)	Density (g/cm^3^)	Porosities
T1	370	1000	0.10	6.36	3.20	5.18	4.422	0.29%
T2	280	1400	0.14	6.19	3.13	5.02	4.396	0.88%
T3	200	500	0.10	5.85	3.06	4.67	4.195	5.41%
